# Social-Cognitive Bias and Depressive Symptoms in Outpatients with Bipolar Disorder

**DOI:** 10.1155/2012/670549

**Published:** 2012-01-23

**Authors:** Guillermo Lahera, Adolfo Benito, Ana González-Barroso, Rocío Guardiola, Sara Herrera, Beatriz Muchada, Noelia Cojedor, Alberto Fernández-Liria

**Affiliations:** ^1^Department of Psychiatry, Príncipe de Asturias University Hospital, University of Alcalá, Carretera Alcalá-Meco s/n, 28805 Alcalá de Henares, Madrid, Spain; ^2^Department of Psychiatry, Hospital Provincial de Toledo, Plaza de la Merced, 4, 45002 Toledo, Spain

## Abstract

A deficit of social cognition in bipolar disorder has been shown, even when patients are stable. This study compares the attribution of intentions (social-cognitive bias) in a group of 37 outpatients with bipolar disorder with 32 matched control subjects. Bipolar patients scored significantly higher in the Ambiguous Intentions Hostility Questionnaire, showing an angry and intentionality bias (*P* = .001, *P* = .02). Differences in blame scale and hostility bias did not reach statistical significance, but a trend was found (*P* = .06). Bipolar patients with depressive symptoms presented a higher score in the angry bias scale (*P* = .03) and aggressivity bias scale (*P* = .004). The global functioning (GAF) correlates significantly with intentionality (*P* = .005), angry (*P* = .027), and aggressivity (*P* = .020) biases. Bipolar patients show a social-cognitive bias that may play a role in their functional outcome.

## 1. Introduction

Attributional style refers to the usual way of explaining why positive and negative events happen to oneself or others. This style has been linked to the development and maintenance of some disorders such as depression [1, 2]. Specifically, depressed patients tend to explain negative events by pessimistic attributions (internal, stable, and global) [3], which is indirectly linked to low self-esteem and low mood [4, 5]. Depression-related dysfunctional attitudes and a negatively biased information processing style have been described in remitted states of bipolar disorder [6, 7]. But other studies have suggested that this dysfunctional cognition in bipolar disorder seems to relate to state rather than to trait [8]. Hypothetically, this attributional style could lead to a social cognitive bias (a distortion of other's intentions) that has not yet assessed in bipolar disorder.

Applied to the field of social cognition, attributional style has been studied in psychotic disorders and it is considered a key domain that involves perceiving, interpreting, and generating responses to others' intentions and behaviours in different situations [9, 10]. In the last decade, several studies have showed an impairment of social cognition in bipolar disorder, both in the symptomatic and in the euthymic phase [11–14]. Most of the papers have studied the “theory of mind” (ToM), defined as “the ability to attribute beliefs, desires, intentions, and emotions to oneself and to others to explain and predict behavior” [15]. But they have not specifically assessed attribution of intentions and its relationship to other clinical or cognitive variables.

The primary objective of this study is to analyse the attribution of intentions (presence or absence of social-cognitive biases) in a group of outpatients with bipolar disorder, compared with a control sample without psychiatric disorders. A secondary objective is to compare the social-cognitive biases of those bipolar patients in state of euthymia with those who present residual depressive symptoms.

## 2. Materials and Methods

### 2.1. Participants

The sample included a total of 69 participants. 37 outpatients fulfilling DSM-IVTR criteria for bipolar disorder (BD) or schizoaffective disorder (SAD) were recruited. Thirty-two healthy comparison subjects without psychiatric or neurologic disorders were also recruited and matched on gender, age, and educational level with the experimental sample.

In the outpatients group, 28 met the criteria for bipolar I disorder, 5 for bipolar II disorder, and 4 for SAD. All patients met criteria for any of the previous diagnoses for at least 3 months. They had been stable and under outpatient followup for at least the 12 previous months. Exclusion criteria were any other psychiatric disorder (axis 1), mental retardation (IQ < 70), organic brain damage, substance abuse/dependence (except nicotine or caffeine dependence), deafness, and difficulties in understanding Spanish. All subjects gave written informed consent to participate in the study. Ethical approval for the study was granted by the Ethics Committee of the Príncipe de Asturias University Hospital.

### 2.2. Instruments

Social cognitive biases were assessed using the Ambiguous Intentions Hostility Questionnaire (AIHQ, [16]). In this task, participants are asked to read 15 vignettes, to imagine the scenario happening to her/him, and to write down the reason why the other person act that way towards him/her. Vignettes include different ambiguous situations, which vary depending on the degree of intentionality of the characters. The subjects are asked to rate on a Likert scale why they think the protagonist acts this way (AIHQHB subscale, Hostility Bias), whether the other person performed the action on purpose (AIHQIS subscale, Intentionality Bias), and how much they would blame him/her (AIHQBS subscale, Blame Scale). Likewise, they rate how angry the situation would make them feel (AIHQAS, Angry Bias) and how they would respond to this situation (AIHQAB, Aggressivity Bias).

Overall functioning status was assessed with two instruments: the Global Assessment Functioning scale (GAF, DSM-IVTR) and Functioning Assessment Short Test (FAST; [17]). The GAF scale assesses the overall performance in Axis V of DSM-IV, with a score between 1 and 100 and includes psychological, social, and occupational operation. The FAST scale identify specific functioning domains such as autonomy, occupational functioning, cognitive functioning, financial issues, interpersonal relationships, and leisure time.

The clinical state of the patients was determined using the Spanish version of the Hamilton Depression Rating Scale (HDRS; [18, 19]) and Young Mania Rating Scale (YMRS; [20, 21]). Regarding depressive symptoms, three breakpoints were established [22, 23]. Euthymic state was defined as HDRS ≤ 7, subsyndromal depression was considered between 8 and 17, and ≥18 was considered like clinical depression. In the YMRS scale were also established three courts: <6 euthymia, 7–14 subsyndromal manic symptoms, and >14 clinical mania [24].  Table 1 shows the sociodemographic data and scores of HDRS and YMRS.

### 2.3. Statistical Analysis

Student's *t*-test and analysis of variance (ANOVA) were used for continuous variables with more than 30 cases and a normal distribution (calculated by Kolmogorov-Smirnov test). If normal adjustment was found, mean and standard deviation were calculated, otherwise median and interquartile range. Qualitative variables were expressed in terms of their absolute and relative frequencies. Nonparametric tests (Mann-Whitney *U* and Kruskal Wallis test) were used for variables that were not normally distributed in the studied population. After the Kruskal Wallis analyses across three groups (bipolar patients in state of euthimia, with subsyndromal depressive symptoms and clinical depressive symptoms), the Mann-Whitney *U* test was used in between-group comparisons of mean values; multiple post hoc comparisons were performed, followed by the calculation of the Bonferroni correction. Pearson correlation was run to identify if depressive symptomatology and global functioning were linked to attributional style. All statistical tests were two tailed, and *P* values were considered significant if =  .05. After Bonferroni correction, *P* value was significant if = 0.017.

## 3. Results

Mean scores in AIHQ subscales were compared between the patient and control groups (Table 2). Bipolar patients scored significantly higher in AIHQAS and AIAQIS subscales (*P* = .001, *P* = .02, resp.). Differences in AIHQBS and AIHQHB did not reach statistical significance, but a trend to a higher score in the bipolar group was also found (*P* = .06).

Subsequently, in order to study the relationship between depressive symptoms and attribution of intentions, we compared the scores on AIHQ subscales between bipolar patients who were euthymic with those with depressive symptoms. As shown in Table 3, significant differences were found in the scales AIHQAS (*P* = .03) and AIHQAB (*P* = .004), along with trends in AIAQHB (*P* = .06) and AIHQBS (*P* = .09).

Influence of depressive symptoms on attribution of intentions may be affected by its severity, so patients were grouped according to the three breakpoints established for HDRS: euthymic ≤ 7 (*n* = 17), with subsyndromal symptoms 8–17 (*n* = 14) and with clinical depression ≥ 18 (*n* = 6). By comparing the 3 groups, significant differences were found on the AIHQAB and AIHQHB subscales (*P* = .04 and *P* = .01, resp.). In order to find in which subgroups there was such a difference, we proceeded to compare two to two by the *U* Mann Whitney test, using the Bonferroni correction (see Table 4). Thus, we found that the subgroup of euthymic patients obtained lower score than patients with subsyndromal depression on the AIHQAB sub-scale (*U* = 59.5, *P* = .017) for the aggression bias. Similarly, the group of euthymic bipolar patients scored lower than those with clinical depression on the AIHQAB and AIHQHB subscales (*U* = 16, *P* = .013 and *U* = 17, *P* = .16, resp.) for the hostility bias (why does the protagonist act like that?) and aggression bias (what would you do in this situation?). However, differences were not found between scores of patients with subsyndromal depression and with clinical depression.

The patient group included a subset of BD type 1 (*n* = 28), type 2 (*n* = 5) and schizoaffective disorder (SAD) (*n* = 4). We reanalyzed the data by subtracting the SAD group (under the hypothesis of an increased attributional bias in the schizophrenic versus bipolar spectrum). Significant differences remained between bipolar patients in euthymia and with depressive symptoms. Thus, scores of euthymic bipolar patients in AIHQHB (*U* = 69, *P* = .045) and AIHQAB (*U* = 58.5, *P* = .014) subscales were lower than those with depressive symptoms. Trend was also maintained in relation to AIHQIS (*U* = 78, *P* = .096) and AIHQAS (*U* = 91, *P* = .068) subscales. In the same way, we reanalysed the differences in the AIHQ Score of bipolar patients in state of euthymia, with subsyndromal depressive symptoms and clinical depression after subtracting the 4 patients diagnosed with SAD, and results are shown in Table 5.

Moreover, we carried out correlation analysis to establish the relationship between the level of global functioning and depressive symptomatology with the performance on the intent attribution test (see Table 6). The global functioning correlates with depressive symptomatology using the GAF scale (*P* = .641; *P* < .000) and in the same way using the FAST scale (*P* = .431; *P* < .000). The global functioning (GAF) correlates significantly with AIHQIS (*P* = .005), AIHQAS (*P* = .027), and AIHQAB (*P* = .020). Using the FAST scale, the correlations did not reach statistical significance.

Finally, we analyzed the possible influence of clinical and subsyndromal manic symptomatology on the intent attribution test. 28 of the 37 patients did not have manic symptoms (YMRS < 7), and 9 of them had subsyndromal symptoms. None met criteria for clinic mania. Nonstatistically significant differences were found in the subscales of AIHQ comparing both groups, AIHQHB (*U* = 106.5; *P* = .953), AIHQIS (*U* = 96.5; *P* = .651), AIHQBS (*U* = 66.5; *P* = .103), AIHQAS (*U* = 98.5; *P* = .709) and AIHQAB (*U* = 100; *P* = .753). In the same way, no significant correlation was found between AIHQ subscales and YMRS scores (see Table 6).

## 4. Discussion

Consistently with previous studies that demonstrated impaired social cognition in the intercritical phase of bipolar disorder, our study shows that these patients have a different attribution of intentions to the general population. Specifically, bipolar outpatients show an intention social-cognitive bias (a higher tendency to attribute intentions to ambiguous scenes) and an anger bias (a tendency to become angry to these situations.) A trend, which could be confirmed in future studies with larger samples, indicates that bipolar patients also have a hostility, guilt, and aggression bias. The second contribution of this study is to show that this attribution of intentions is significantly influenced by the presence of depressive symptoms. Thus, bipolar patients who presented residual depressive symptoms showed a higher social-cognitive bias compared to those who maintained euthymic, particularly anger, and aggression bias.

When attribution of intentions between bipolar patients with clinical and subsyndromal depressive symptoms was compared; differences were not found. But differences between these two groups and patients in euthymic state were found. All of this highlights the critical importance of depressive symptoms (whatever the intensity) in the attribution of intentions to everyday scenes. Accordingly, results show that subsyndromal manic symptoms are not so decisive in this domain of social cognition as depressive symptoms.

Depressive symptoms in the intercritical phase of bipolar disorder are very common [25–28]. The influence of depressive symptoms in the functional outcome of patients has been reported in several studies, showing that it seems a strong predictor of functional outcome, together with cognitive variables such as verbal memory and executive functions [29, 30]. Recently, a study has found out that the presence of even mild depressive symptoms significantly interfere in the recovery of patients after an episode of bipolar mania [31]. Thus, it appears that depressive symptoms should be incorporated into an explicative model of the functional outcome of bipolar disorder, along with other clinical and cognitive variables [32]. The contribution of our study is to point out that depressive symptomatology seems to have a decisive influence on a relevant variable such as attribution of intentions–considered a key domain of social cognition [10], which is altered in bipolar patients. Along the same line as previous studies [33], our findings suggest that social cognition may be a mediator between affective symptomatology and psychosocial outcome in bipolar disorder.

This study has various limitations which should be taken into account when the results are interpreted. First, the sample was relatively small and heterogeneous. Despite this fact, when analyzing the data by subtracting the group diagnosed with schizoaffective disorder (under the hypothesis of an enhanced attributional bias in schizophrenia versus bipolar spectrum), significant differences remain between the euthymia and depression groups, again on the hostility and Aggressivity bias. Another limitation is the potential interference of psychotropic drugs on the performance of the tests, since all patients were on pharmacological treatment.

## 5. Conclusions

Bipolar outpatients show an intention social-cognitive bias compared to healthy controls. This bias is significantly influenced by the presence of residual depressive symptoms.

Future studies should analyze the different variables involved in functional outcome of bipolar disorder, such as cognitive function, clinical variables (with special emphasis on subsyndromal depressive symptoms), and, as suggested in this study, social-cognitive biases.

## Figures and Tables

**Table 1:** Sociodemographic characteristics of the sample.

	Patients	Controls		
Age	38.73 (11.6)	44.22 (12.6)	*t*-test = −1.88	*P* = .06
% Female	64.8%	68.7%	*χ*_(1)_^2^ = .18	*P* = .73
% Education level				
Primary	29.7% (*n* = 11)	25% (*n* = 8)	*χ*_(3)_^2^ = 2.15	*P* = .54
Secondary	48.6% (*n* = 18)	40.6% (*n* = 13)		
University	21.6% (*n* = 8)	34.4% (*n* = 11)	*χ*_(3)_^2^ = 4.9	*P* = .18
% Civil state				
Single	43.2% (*n* = 16)	40.6% (*n* = 13)		
Married	29.7% (*n* = 11)	50% (*n* = 16)		
Divorced	24.3% (*n* = 9)	9.4% (*n* = 3)		
Widowed	2.7% (*n* = 1)	0% (*n* = 0)		

Hamilton scale	8.42 (7.38)	.19 (1.06)	*t*-test = 6.61	*P* ≤ .00
Young scale	4.47 (4.94)	0 (0)	*t*-test = 5.43	*P* ≤ .00

**Table 2:** Scores in the AIHQ subscales of the bipolar patients and healthy controls.

AIHQ	Patients		Controls		
	*N* = 37		*N* = 32		
	M	SD	M	SD	*t* Student (*P*)
AIHQHB	1.84	.53	1.62	.43	1.89 (.06)
AIHQIS	1.84	.73	2.69	.60	3.32* (.00)
AIHQAS	2.87	.76	2.47	.65	2.34* (.02)
AIHQBS	2.83	.74	2.58	.76	1.39 (.17)
AIHQAB	1.92	.63	1.67	.48	1.88 (.06)

*Statistically significant differences (*P* < .05).

**Table 3:** Scores in the AIHQ subscales of the bipolar patients in state of euthymia and those with depressive symptoms.

AIAQ	Euthymia	Depressive symptoms	*U* mann whitney
	*n* = 17	*n* = 20	
	M (SD)	M (SD)	*U* (*P*)
AIAQHB	1.63 (.37)	2.02 (.61)	108 (.06)
AIAQIS	3.09 (.45)	3.36 (.91)	116 (.10)
AIAQAS	2.61 (.78)	3.09 (.70)	99.5 (.03)*
AIAQBS	2.62 (.68)	3.01 (.76)	114.5 (.09)
AIAQAB	1.59 (.37)	2.21 (.67)	76.5 (.004)*

*Statistically significant differences (*P* < .05).

**Table 4:** Scores in the AIHQ subscales of the bipolar patients in state of euthymia, with subsyndromal depressive symptoms and clinical depression.

AIHQ	Euthymia^1^ *n* = 17	Subsyndromal depression^2^ *n* = 14	Clinical depression^3^ *n* = 6	Kruskal wallis	*U* Mann whitney
	M (SD)	M (SD)	M (SD)	*χ*_(2)_^2^ (*P*)	*U* (*P*)
AIHQHB	1.63 (.37)	1.87 (.57)	2.36 (.55)	6.44 (.04)*	^1–3^16 (.01)** ^1-2^92 (.29) ^2-3^21 (.09)
AIHQIS	3.09 (.45)	3.21 (1)	3.7 (.56)	3.97 (.14)	^1–3^20 (.03) ^1-2^96 (.37) ^2-3^32 (.44)
AIHQAS	2.61 (.78)	2.99 (.71)	3.31 (.64)	5.11 (.08)	^1–3^24 (.06) ^1-2^75.5 (.08) ^2-3^32.5 (.44)
AIHQBS	2.62 (.68)	2.89 (.82)	3.28 (.59)	4.18 (.12)	^1–3^20 (.03) ^1-2^94.5 (.34) ^2-3^31 (.4)
AIHQAB	1.59 (.37)	2.13 (.68)	2.39 (.66)	8.67 (.01)*	^1–3^17 (.016)** ^1-2^59.5 (.017)** ^2-3^32 (.44)

1–3 = comparison euthymia—clinical depression; 1-2 = comparison euthymia—subsyndromal depressive symptoms; 2-3 = comparison subsyndromal depressive symptoms—clinical depression. (*P ≤ .05; **P ≤ .017 for Bonferroni correction.)

**Table 5:** Scores in the AIHQ subscales of the bipolar patients in state of euthymia, with subsyndromal depressive symptoms and clinical depression (after subtracting patients with SAD, *n* = 4).

AIHQ	Euthymia^1^ *n* = 15	Subsyndromal depression^2^ *n* = 13	Clinical depression^3^ *n* = 5	Kruskal wallis	*U* Mann whitney
	M (SD)	M (SD)	M (SD)	*χ*_(2)_^2^ (*P*)	*U* (*P*)
AIHQHB	24.87 (5.79)	29.08 (8.09)	34 (8.51)	4.54 (.10)	^1–3^15 (.05) ^1-2^69.5 (.2) ^2-3^21 (.29)
AIHQIS	47 (6.56)	48.85 (15.22)	53.6 (7.92)	2.58 (.28)	^1–3^19 (.12) ^1-2^74 (.29) ^2-3^30 (.85)
AIHQAS	40.33 (11.47)	44.77 (11.12)	49.2 (10.76)	3.11 (.21)	^1–3^22 (.2) ^1-2^66 (.16) ^2-3^26.5 (.57)
AIHQBS	41 (8.71)	42.23 (11.91)	49 (9.92)	2.6 (.27)	^1–3^18 (.1) ^1-2^88 (.68) ^2-3^21.5 (.29)
AIHQAB	24.4 (5.68)	32.54 (10.35)	33.6 (9.21)	6.22 (.04)	^1–3^16.5 (.07) ^1-2^50 (.03) ^2-3^30 (.85)

1–3 = comparison euthymia—clinical depression; 1-2 = comparison euthymia—subsyndromal depressive symptoms; 2-3  = comparison subsyndromal depressive symptoms—clinical depression. (*P ≤ .05; **P ≤ .017 for Bonferroni correction.)

**Table 6:** Correlation between global functioning (GAF, FAST) and depressive symptomatology with scores in the AIHQ subscales.

		AIAQHB	AIAQIS	AIAQAS	AIAQ	AIAQAB	GAF	FAST	HAM-D	YMRS
GAF	Pearson	−.216	−.334**	−.268*	−.154	−.281*	1			
	Sig. (bilateral)	.077	.005	.027	.209	.020				

FAST	Pearson	.121	.228	.068	.057	.187	−.434**	1
	Sig. (bilateral)	.320	.059	.578	.642	.123	.000			

HAM-D	Pearson	.364**	.339**	.316**	**.196**	.436**	−.641**	.431**	**1**	
	Sig. (bilateral)	**.002**	**.005**	**.009**	**.109**	**.000**	**.000**	**.000**		

YMRS	Pearson	.012	.145	.135	−.049	.033	−.478**	.257**	.19	1
	Sig. (bilateral)	.923	.237	.272	.693	.786	.000	.034	.12	

**Significant correlation at level  .01 (bilateral).

*Significant correlation at level  .05 (bilateral).
